# Editorial: Molecular physiology of tissue adaptation to acute ischemic injury

**DOI:** 10.3389/fphys.2023.1179083

**Published:** 2023-03-14

**Authors:** Egor Plotnikov, Andrei Krivtsov, Aleksey Ustyugov

**Affiliations:** ^1^ A.N. Belozersky Institute of Physico-Chemical Biology, Lomonosov Moscow State University, Moscow, Russia; ^2^ Department of Pediatric Oncology, Dana Farber Cancer Institute, Harvard Medical School, Boston, MA, United States; ^3^ Institute of Physiologically Active Compounds, Federal Research Center of Problems of Chemical Physics and Medicinal Chemistry of the RAS, Chernogolovka, Russia

**Keywords:** ischemia, kidney, heart, mesenchymal stem cells, nicorandil, hub genes, white adipose tissue, osthole

Ischemia is a condition of reduced blood flow to tissues, which is often followed by a detrimental reperfusion process upon restoration of blood flow (Wu et al., 2018). Ischemia/reperfusion (I/R) injury can affect any organ and lead to critical pathological conditions such as ischemic stroke, myocardial infarction (MI), acute kidney injury (AKI), and others (Eltzschig and Eckle, 2011). Despite active research in this field, not all the molecular mechanisms and consequences of I/R have been fully disclosed, which impedes the development of effective and timely treatments.

One of the most pronounced effects of I/R injury is the change in gene expression in the cells of damaged tissue. Regarding this, He et al. performed comprehensive bioinformatic analysis of two datasets containing mRNA expression profiles of kidney cells after I/R exposure. The study revealed that the majority of differentially expressed genes were upregulated in the I/R group and reflected the processes of inflammation and apoptosis. Bioinformatic analysis found ten hub genes, including *Jun, Stat3, MYC, Cdkn1a, Hif1a, FOS, Atf3, Mdm2, Egr1,* and *Ddit3*, which may become useful biomarkers of AKI at the early stages. Interaction networks were also constructed, which helped to predict transcription factors and mi-RNAs that may interact with discovered hub genes and signaling pathways. Moreover, authors proposed molecular compounds that may be efficient in the therapy of AKI.

Since the lack of appropriate therapy of ischemic pathologies, new approaches and drugs are being actively developed. Osthole is suggested as a promising drug for AKI treatment. Osthole is a natural coumarin from *Cnidium monnieri*, which has proven its anti-inflammatory, immunomodulatory, and anti-cancer effects in different organs by regulating PI3K/Akt signaling pathway. Dai et al. showed that osthole inhibited HMGB1 gene transcription and protein synthesis, reduced its acetylation and release from the nucleus into cytoplasm and extracellular space, which may be the main reason for its nephroprotective effects during renal I/R.

A dangerous complication of ischemic pathologies is that I/R injury of one organ can lead to multi-organ damage. For instance, ischemic AKI may provoke pancreatic injury and disrupt insulin production by causing the death of beta cells. To preserve the functioning of pancreas during renal I/R, ShamsEldeen et al. tested therapy based on bone marrow mesenchymal stem cells and nicorandil administration. Nicorandil is a mitochondrial K-ATP channel opener, which also acts as nitric oxide donor. Opening of K-ATP channels by nicorandil may lead to hyperpolarization of the beta cells and prevent insulin release during renal I/R-induced pancreatic injury. Indeed, combined systemic nicorandil administration and nicorandil-preconditioned mesenchymal stem cells maintained the most pronounced survival of pancreatic tissue and ameliorated apoptosis and inflammation through activation of PI3K/Akt/mTOR signaling pathway.

Multi-organ damage is also a common consequence of MI. Wang et al. observed the influence of cardiac I/R injury on visceral and subcutaneous white adipose tissue. Since adipose tissue is an important endocrine organ that secretes a range of adipokines, cytokines, and micro-RNAs, its functioning during and after ischemic exposure is of great interest. To unravel the effects of MI on fat depots, authors monitored morphology, cellular infiltrates, and gene expression of adipocyte cells for 28 days post ischemia. They demonstrated that cardiac I/R led to a decrease in adipocyte size in subcutaneous, but not visceral white adipose tissue, indicating more pronounced susceptibility of subcutaneous fat depots to MI. Moreover, after cardiac I/R, subcutaneous adipose tissue showed reprogramming to brown-like phenotype, higher macrophage infiltration, and a reduction in adipokine gene expression.

## Conclusion

Diseases associated with I/R injury are characterized by high morbidity, mortality, and healthcare costs and represent a global public health concern. The articles collected in the Research Topic aimed to gain an understanding of molecular mechanism of renal and cardiac I/R, as well as to identify potential molecular markers and drug targets for the treatment. Eventually, the studies found hub genes activated during ischemic injury, discovered osthole and nicorandil as promising therapeutic compounds, analyzed kidney–pancreas and heart–adipose tissue crosstalks. We hope that in-depth research based on these findings will open up new therapeutic strategies in the prevention and treatment of ischemic pathologies.
